# RACE: A Modular and Interpretable Radial–Angular Correlation Descriptor for Atomistic Machine Learning

**DOI:** 10.1021/acs.jctc.6c01111

**Published:** 2026-10-08

**Authors:** Raphael M. Tromer, Jhionathan de Lima, Cristiano Francisco Woellner, Paola Ferreira Barbosa, Roberto Ventura Santos, Luiz Antonio Ribeiro Junior

**Affiliations:** † University of Brasília, Institute of Physics, Brasília, Federal District 70910-900, Brazil; ‡ Department of Physics, 28122 Federal University of Paraná, 81531-980 Curitiba, PR, Brazil; § Interdisciplinary Center for Science, Technology, and Innovation (CICTI), Federal University of Paraná, 81530-000 Curitiba, PR, Brazil; ∥ Institute of Geosciences, University of Brasília, Brasília 70910-900, DF, Brazil; ⊥ Department of Physics, 28127 Norwegian University of Science and Technology (NTNU), NO-7491 Trondheim, Norway; # Computational Materials Laboratory, LCCMat, Institute of Physics, 8018 University of Brasília, 70910-900 Brasília, Federal District, Brazil

## Abstract

Atomistic machine learning (ML) representations involve trade-offs among predictive accuracy, physical interpretability, descriptor dimensionality, and computational cost. Here, we introduce the Radial–Angular Correlation Descriptor (RACE), a modular representation constructed from radial distributions, angular distributions, and an explicit joint radial–angular map. Chemical identity is incorporated through interchangeable scalar or multichannel weighting functions rather than through a mandatory set of element-pair channels. We evaluate RACE against conventional smooth overlap of atomic positions (SOAP), compressed SOAP-μ_2_, atom-centered symmetry functions (ACSF), and a fixed-basis atomic cluster expansion (ACE) representation using common train–test partitions, training-only feature selection, repeated evaluations, and uncertainty estimates. RACE provides useful predictive accuracy across structurally and chemically diverse systems, but it does not uniformly outperform the alternative representations. Compressed SOAP is particularly accurate for the chemically heterogeneous benchmarks, whereas explicit composition features are required for robust generalization across broad Materials Project chemical spaces. Direct ablation of the radial, angular, and joint channels further shows that the incremental value of the joint radial–angular term is system dependent. The principal practical characteristics of RACE are therefore its transparent component structure, configurable chemical encoding, descriptor dimension independent of the number of species in its scalar-weighted form, and low peak memory during descriptor generation. RACE is consequently presented as a simple and manipulable structural representation that complements, rather than universally replaces, systematic density-correlation descriptors.

## Graphical abstract

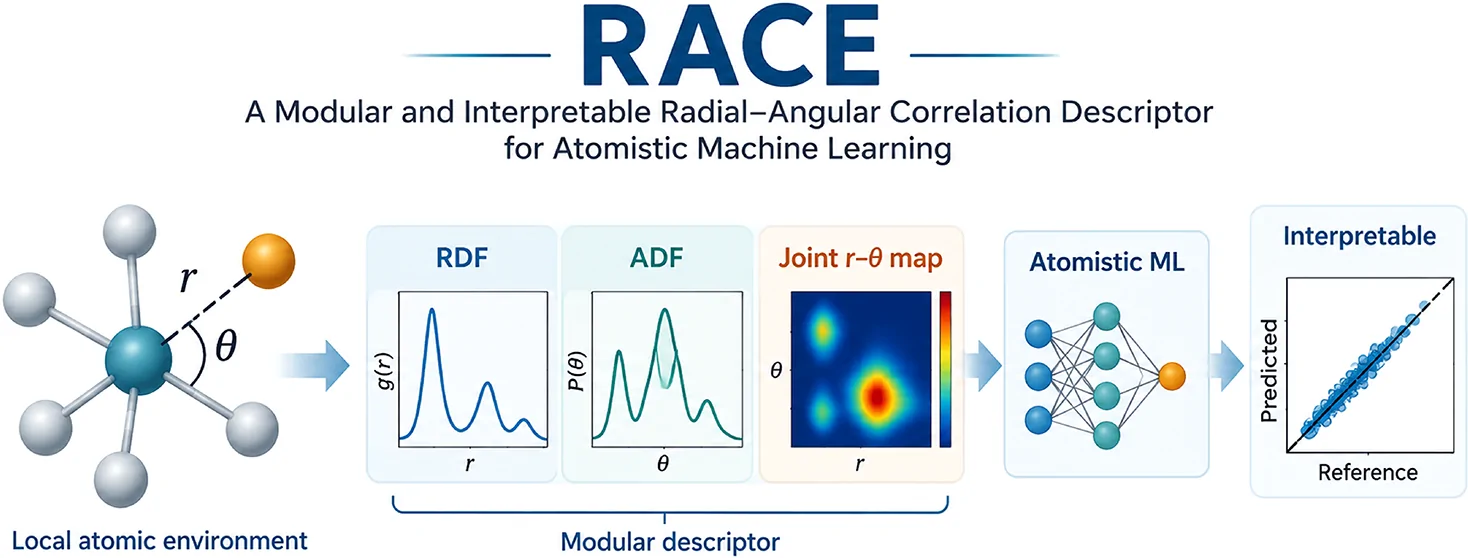

## Introduction

Machine learning (ML) has become an increasingly important component of atomistic and materials modeling, enabling the prediction of energetic, electronic, mechanical, and thermodynamic properties across chemical and configurational spaces that are difficult to sample exhaustively using first-principles calculations alone.^1–5^ Applications range from formation energy prediction and phase-stability screening^6,7^ to machine learning interatomic potentials^8,9^ and data-driven materials discovery.^10–12^ A central modeling decision in each of these applications is the representation of atomistic structure because it determines which geometric and chemical relationships are exposed to the learning algorithm.^13–16^

A useful structural representation should respect translational, rotational, and permutation symmetries, vary smoothly under small displacements, retain the information relevant to the target property, and remain computationally manageable for the intended data set.^13,17^ No single descriptor is optimal under all of these criteria. Compact representations may be transparent and inexpensive but can depend sensitively on parametrization. Systematic density or body-order expansions can provide high structural resolution, but their native feature spaces and model construction may be more demanding.

Atom-centered symmetry functions (ACSF) encode local radial and angular environments through predefined invariant functions.^9,16,18^ The smooth overlap of atomic positions (SOAP) representation expands a smooth neighbor density in radial basis functions and spherical harmonics before constructing rotational invariants.^13,19^ Atomic cluster expansion (ACE) methods provide systematic body-ordered invariant or equivariant bases.^20^ Learned graph and equivariant representations constitute a further class of methods that can optimize chemical and geometric features directly from data.^8,10^ The present study is restricted to fixed structure-level representations under a common tree-based regression protocol and does not claim a general comparison with optimized learned equivariant architectures.

The treatment of chemical identity requires particular care. In conventional element-resolved SOAP, distinct species are assigned separate density channels, and the number of power-spectrum terms can increase rapidly with the chemical alphabet. However, alchemical kernels, low-rank chemical maps, compressed SOAP formulations, descriptor reduction, and supervised feature selection strategies can substantially mitigate this growth.^21–23^ Recent applications of alchemical compression to high-entropy alloys further demonstrate that compact chemical representations can remain effective across large elemental alphabets,^24,25^ while continuous alchemical degrees of freedom have also been introduced in learned interatomic potentials.^26^ This manuscript therefore does not describe SOAP as intrinsically unsuitable for multicomponent systems, nor does it claim chemical compression as unique to RACE. Instead, it distinguishes conventional element-channel SOAP from compressed formulations and evaluates both explicitly.

The motivation for RACE is consequently not that radial or angular correlations are fundamentally new, nor that chemical compression is absent from the literature. RACE is designed as an explicit and directly manipulable radial–angular representation. It separates a local environment into three numerical objects: a radial distribution function (RDF), an angular distribution function (ADF), and a joint radial–angular map *F*(*r*, θ). These channels can be visualized, removed, concatenated, or assigned alternative chemical maps without reformulating a complete density expansion.

The joint term is conceptually related to three-body information represented within SOAP power spectra, ACE bases, and other body-ordered descriptors. It is not presented here as a new formal invariant or as a complete many-body basis. Its practical role is to expose where particular distance and angle motifs occur together. This organization facilitates component-level interpretation and controlled ablation, even when another representation provides lower prediction error.

Chemical identity is introduced through a general elemental map ϕ*_c_*, where *c* denotes one or more chemical channels. The map may be constant, based on atomic number, electronegativity, covalent radius, or valence-electron count, or obtained by concatenating multiple channels. Atomic-number weighting is therefore treated as a parameter-free default and not as a uniquely physical measure of chemical similarity. The present work evaluates RACE across a hierarchy of benchmark systems that progressively increase in structural and chemical complexity. The analysis is designed to separate several distinct questions that are often conflated in descriptor assessment: predictive accuracy in chemically simple systems, the independent contribution of radial, angular, and joint radial–angular terms, the effect of chemical encoding, the role of explicit composition in broad chemical spaces, and the computational consequences of descriptor construction and dimensionality.

To this end, RACE is compared with conventional SOAP, compressed SOAP-μ_2_, ACSF, and a fixed-basis ACE representation under a common machine learning protocol. Repeated data partitions, training-only feature selection, uncertainty estimates, grouped validation strategies, and direct component ablations are used to distinguish robust trends from partition-dependent fluctuations. The resulting analysis is intended to clarify the practical regime of applicability of RACE rather than to establish a universal ranking among descriptors. In particular, we examine whether the explicit radial–angular organization provides useful interpretability and competitive predictive performance, how sensitive the representation is to the chosen chemical map, and which aspects of its computational behavior are genuinely advantageous relative to established alternatives.

## Methodology

### RACE Descriptor Formulation

For a central atom *i*, the local environment contains all neighbors *j* satisfying *r*_*ij*_ < *R*_*c*_. Neighbor contributions are smoothly attenuated by

1 $$w_{c}\left(r_{\mathrm{ij}}\right)=\{\begin{array}{ll} \frac{1}{2}\left[\cos\left(\frac{\pi r_{\mathrm{ij}}}{R_{c}}\right)+1\right], & r_{\mathrm{ij}}<R_{c},\\ 0, & r_{\mathrm{ij}}\geq R_{c} \end{array}$$

Equation 1 ensures that neighbor contributions vanish smoothly at the cutoff radius.

Chemical identity is introduced through one or more scalar maps ϕ_*c*_(*Z*). The pair weight in channel *c* is

2 $$w_{\mathrm{ij}}^{\left(c\right)}=w_{c}\left(r_{\mathrm{ij}}\right)\phi_{c}\left(Z_{i}\right)\phi_{c}\left(Z_{j}\right)$$

Equation 2 separates the geometric cutoff from the selected chemical weighting. The geometry-only representation uses ϕ­(*Z*) = 1. The atomic-number channel uses ϕ_*Z*_(*Z*) ∝ √*Z*, recovering the $\sqrt{Z_{i}Z_{j}}$ factor used in the original implementation. Alternative channels use normalized electronegativity, covalent radius, or valence-electron count. The multichannel representation concatenates the vectors obtained from the selected scalar maps. Scalar-weighted RACE therefore has a dimension independent of the number of species, whereas the multichannel variant grows linearly with the number of selected chemical maps.

This construction differs from conventional element-resolved SOAP in where chemical identity enters the representation. In conventional SOAP, separate species-resolved neighbor densities are expanded before rotational invariants are formed, whereas scalar RACE multiplies the same explicit radial and angular kernels by a low-dimensional elemental map. Consequently, the single-channel RACE grid does not acquire a separate block for each species or species pair. This is an organizational distinction rather than a unique scalability mechanism: compressed SOAP and other alchemical mappings likewise reduce chemical-channel growth, but retain a density expansion construction.

The radial channel is

3 $${\mathrm{RDF}}_{p}^{\left(i,c\right)}=\sum_{j}w_{\mathrm{ij}}^{\left(c\right)}\exp\left[-\frac{{\left(r_{\mathrm{ij}}-\mu_{p}\right)}^{2}}{2\sigma_{r}^{2}}\right]$$

Equation 3 accumulates the chemically weighted neighbor density around each radial center μ_*p*_. The angular channel is

4 $${\mathrm{ADF}}_{q}^{\left(i,c\right)}=\sum_{j<l}w_{\mathrm{ij}}^{\left(c\right)}w_{\mathrm{il}}^{\left(c\right)}\exp\left[-\frac{{\left(\theta_{\mathrm{jil}}-\nu_{q}\right)}^{2}}{2\sigma_{\theta }^{2}}\right]$$

Equation 4 analogously resolves neighbor-pair angles around the centers ν_*q*_. The joint channel is

5 $$F_{p,q}^{\left(i,c\right)}=\sum_{j<l}w_{\mathrm{ij}}^{\left(c\right)}w_{\mathrm{il}}^{\left(c\right)}\left[K_{r}\left(r_{\mathrm{ij}};\mu_{p}\right)+K_{r}\left(r_{\mathrm{il}};\mu_{p}\right)\right]K_{\theta }\left(\theta_{\mathrm{jil}};\nu_{q}\right)$$

with

6 $$K_{r}\left(r;\mu_{p}\right)=\exp\left[-\frac{{\left(r-\mu_{p}\right)}^{2}}{2\sigma_{r}^{2}}\right]$$

and

7 $$K_{\theta }\left(\theta ;\nu_{q}\right)=\exp\left[-\frac{{\left(\theta -\nu_{q}\right)}^{2}}{2\sigma_{\theta }^{2}}\right]$$

Equations 5, ([Disp-formula eq6]), and ([Disp-formula eq7]) define the explicit radial–angular correlation channel and its Gaussian kernels.

The local vector is

8 $$x^{\left(i,c\right)}=\left[{\mathrm{RDF}}^{\left(i,c\right)},{\mathrm{ADF}}^{\left(i,c\right)},F^{\left(i,c\right)}\right]$$

Equation 8 concatenates the radial, angular, and joint local channels. A structure-level vector is obtained by concatenating the mean and standard deviation of every local component

9 $$X_{\mathrm{RACE}}^{\left(c\right)}=\left[{\left\langle x^{\left(i,c\right)}\right\rangle }_{i},{\mathrm{std}}_{i}\left(x^{\left(i,c\right)}\right)\right]$$

Equation 9 converts the atom-centered vectors into a fixed-length structure-level representation through mean and standard deviation pooling. For one chemical-channel

10 $$D_{\mathrm{RACE}}=2N_{R}+2N_{A}+2N_{R}N_{A}$$

Equation 10 gives the resulting feature dimension for one chemical-channel. With *N*_R_ = *N*_A_ = 16, the RDF, ADF, RDF+ADF, joint-only, and full-RACE representations contain 32, 32, 64, 512, and 576 features, respectively.

Distances and angles provide translational and rotational invariance, and sums over neighbors and neighbor pairs provide permutation invariance. The cutoff and Gaussian kernels produce continuous variation under atomic displacements. RACE is a finite-resolution descriptor and is not claimed to be complete or systematically improvable in the formal sense of SOAP or ACE.

### RACE Parameters

Unless stated otherwise, the calculations use the common parameters in Table 1. Holding these values fixed avoids data set-specific tuning.

**Table 1:** Default Parameters of the Single-Channel RACE Representation

Parameter	Symbol	Value
Cutoff radius	*R*_c_	4.5 Å
Minimum radial center	*r*_min_	1.0 Å
Radial centers	*N*_R_	16
Angular centers	*N*_A_	16
Radial Gaussian width	σ_r_	0.08 Å
Angular Gaussian width	σ_θ_	7°
Structure aggregation	—	mean and standard deviation

The common cutoff of 4.5 Å was selected as a fixed compromise across the benchmark set: it includes the near-neighbor environment and additional local coordination structure while limiting the neighbor-pair growth of the angular and joint channels. The same cutoff is used for the reference descriptors where applicable so that cutoff range is not an independently tuned source of performance differences. We do not regard 4.5 Å as universally optimal; holding it fixed is intended to avoid data set-specific hyperparameter tuning.

The joint block grows as *N*_R_ *N*_*A*_. Thus, although the scalar-weighted descriptor dimension is independent of the number of species, it is not independent of geometric resolution. The ablation and scalability analyses explicitly address this distinction.

### Reference Representations

All local descriptors were converted to one structure-level vector by concatenating their atomic means and standard deviations, unless an implementation directly returned a global vector.

Conventional SOAP was generated with *r*_cut_ = 4.5 Å, *n*_max_ = 8, *l*_max_ = 6, Gaussian width σ = 0.3 Å, and explicit species channels. Compressed SOAP-μ_2_ uses the same geometric hyperparameters and a normalized square-root atomic-number species map. This preserves a continuous chemical signal while keeping the compressed representation independent of the number of species.

ACSF was generated using a 4.5Å cutoff and fixed radial and angular parameter lists. For the carbon benchmarks, the radial functions use (η, *R*_*s*_) = (1, 0.5), (1, 1.0), (1, 2.0), and the angular functions use (η, ζ, λ) = (1, 1, 1), (1, 2, 1). The full multicomponent parameter definitions are provided in the Supporting Information.

ACE results correspond to a fixed, untrained basis representation and not to a separately optimized ACE interatomic potential. The basis uses a 4.5 Å cutoff, a Chebyshev-type radial basis, and a finite body-order and angular truncation. The complete basis specification and resulting dimensions are reported in the Supporting Information. We therefore use “ACE representation” in this restricted sense.

The native dimensions differ across descriptors. For Figure 2 and Figure 4, at most 50 features are selected inside each training partition, providing a common upper bound on the effective input dimension. The feature selector is never fitted on held-out data. Figure 3 is a component-removal ablation and uses the native dimension of each RACE variant. Figure 7 reports native descriptor dimension separately from downstream model cost.

### Data Sets and Target Properties

The first benchmark contains 120 two-dimensional (2D) carbon allotropes reported by Shi et al.^27^ The structures share one chemical species but contain different ring topologies and local sp^2^ motifs. The target is the corresponding structure energy in eV.

The vacancy-defective graphene benchmark was generated from 288-atom graphene supercells by removing atoms at nominal concentrations of 5%, 10%, and 15%. Fifty independent configurations were retained at each concentration, giving 150 structures. Reference values were obtained with DFTB+.^28–30^ The target is the defect-related energy in eV, and repeated partitions are stratified by vacancy concentration.

The distorted diamond benchmark contains 250 configurations derived from a 54-atom diamond supercell. Random displacements of up to 0.15 Å were applied without changing composition or topology, and DFT energies were used as targets. The purpose of this controlled off-equilibrium benchmark is to isolate sensitivity to smooth local geometric perturbations without simultaneously changing chemistry or bonding topology. ”Distorted” therefore denotes displaced configurations of the same diamond phase rather than a distinct structural phase.

The periodic chemically complex benchmark is an FCC Co–Fe–Cu–Ni–Pt high-entropy alloy containing 250 decorrelated distorted configurations. The finite benchmark is a 55-atom icosahedral nanoparticle with nominal composition Al_70_Co_10_Fe_5_Ni_10_Cu_5_ and 250 decorrelated configurations.^31^ Because Co- and Fe-containing alloys can exhibit magnetic degrees of freedom, we emphasize that the RACE representation used here contains nuclear geometry and elemental maps only. It does not encode spin configurations or local magnetic moments. The HEA benchmark therefore tests structural and chemical encoding, not transferability between distinct magnetic states.

The Materials Project benchmark is a curated heterogeneous subset spanning multiple reduced formulas and chemical systems.^32^ Formation energy per atom is used as the regression target so that the model is not trained on an extensive total energy that changes trivially with cell size. The final curated subset contains 250 structures. The validation split sizes and chemical-system grouping statistics are reported in the Supporting Information. Duplicate entries and structures lacking the required target or grouping metadata were removed. Together with the five-component HEA and nanoparticle benchmarks, this data set probes chemical heterogeneity; the separate scaling experiments in Figure 7 test dependence on species count and atom-count and should not be interpreted as a claim of universal large-system superiority.

### Machine Learning and Statistical Protocol

Extreme Gradient Boosting regression was used for all fixed-descriptor comparisons.^33^ Unless otherwise specified, the final model uses 500 trees, learning rate 0.05, maximum depth 5, unit row and column subsampling, *L*_2_ regularization of 1, and the histogram tree method. A separate 300-tree XGBoost model ranks features using only the training subset. Constant training features are removed before ranking, and at most 50 features are passed to the final model.

Figure 2, Figure 4, and Figure 5 use 20 repeated shuffled train–test partitions for test fractions from 0.1 to 0.8. Within a data set and test fraction, every descriptor uses identical training and test indices. Figure 3 uses 20 repeated 80/20 partitions. Curves report the mean over partitions and shaded regions report the 95% confidence interval

11 $${\mathrm{CI}}_{95}=1.96\frac{s}{\sqrt{n}}$$

Equation 11 is used for all 95% confidence intervals reported from repeated partitions, where *s* is the standard deviation across *n* = 20 repeats.

The principal metrics are

12 $$\mathrm{MAE}=\frac{1}{N_{\mathrm{test}}}\sum_{i}\left|y_{i}-{ŷ}_{i}\right|$$

and

13 $$\mathrm{RMSE}=\sqrt{\frac{1}{N_{\mathrm{test}}}\sum_{i}{\left(y_{i}-{ŷ}_{i}\right)}^{2}}$$

Equations 12 and ([Disp-formula eq13]) define the two principal error metrics used throughout the manuscript. The coefficient of determination is reported in the Supporting Information, but it is not used as the primary measure of predictive quality. Out-of-fold parity plots and prediction tables are also provided in the Supporting Information.

Paired comparisons use identical partition seeds. Two-sided Wilcoxon signed-rank tests are applied to paired errors and corrected using the Holm procedure within each comparison family. Statistical significance is interpreted together with effect magnitude and direction.

### Materials Project Grouped Validation and Composition Baselines

Three validation protocols are used for Materials Project. Random partitions measure interpolation within the sampled database. Reduced-formula grouping prevents the same reduced stoichiometry from appearing in training and test sets. Chemical-system grouping prevents structures containing the same set of elements from appearing on both sides of a partition. Grouped validation is performed only when at least two distinct groups are available. Small group sets use leave-one-group-out evaluation.

The composition-only baseline is the normalized elemental-fraction vector. RACE + composition concatenates this vector with RACE. These conditions separate local structural information from global stoichiometric information. SOAP-μ_2_ and ACE are evaluated using the same group assignments.

### Computational Benchmarking

Descriptor generation was measured in a fresh process using one CPU thread. Peak memory is the maximum resident set size of the complete process tree. Native dimensionality, CSV storage, dense-matrix storage, generation time per structure, and feature selection plus model training time were measured separately.

The representative benchmark uses a balanced batch. Species scaling is measured at approximately constant atom-count by varying the chemical alphabet with approximately 4% substitution. Atom-count scaling uses pure-carbon structures, preventing chemical-channel growth from being conflated with system size. Error bars are 95% confidence intervals across repeated measurements. Hardware, software versions, repetition counts, and numerical storage precision are documented in the Supporting Information.

## Results and Discussion

### RACE Descriptor Representation and Physical Interpretation

Figure 1 shows how the RACE descriptor encodes structural information in representative atomistic systems. For each structure, three complementary components are displayed: the radial distribution function (RDF), the angular distribution function (ADF), and the joint radial–angular correlation map *F*(*r*, θ). These components provide a direct physical interpretation of the descriptor by resolving pairwise distance distributions, bond-angle statistics, and the coupling between radial and angular motifs within local atomic environments.

**Figure 1:**
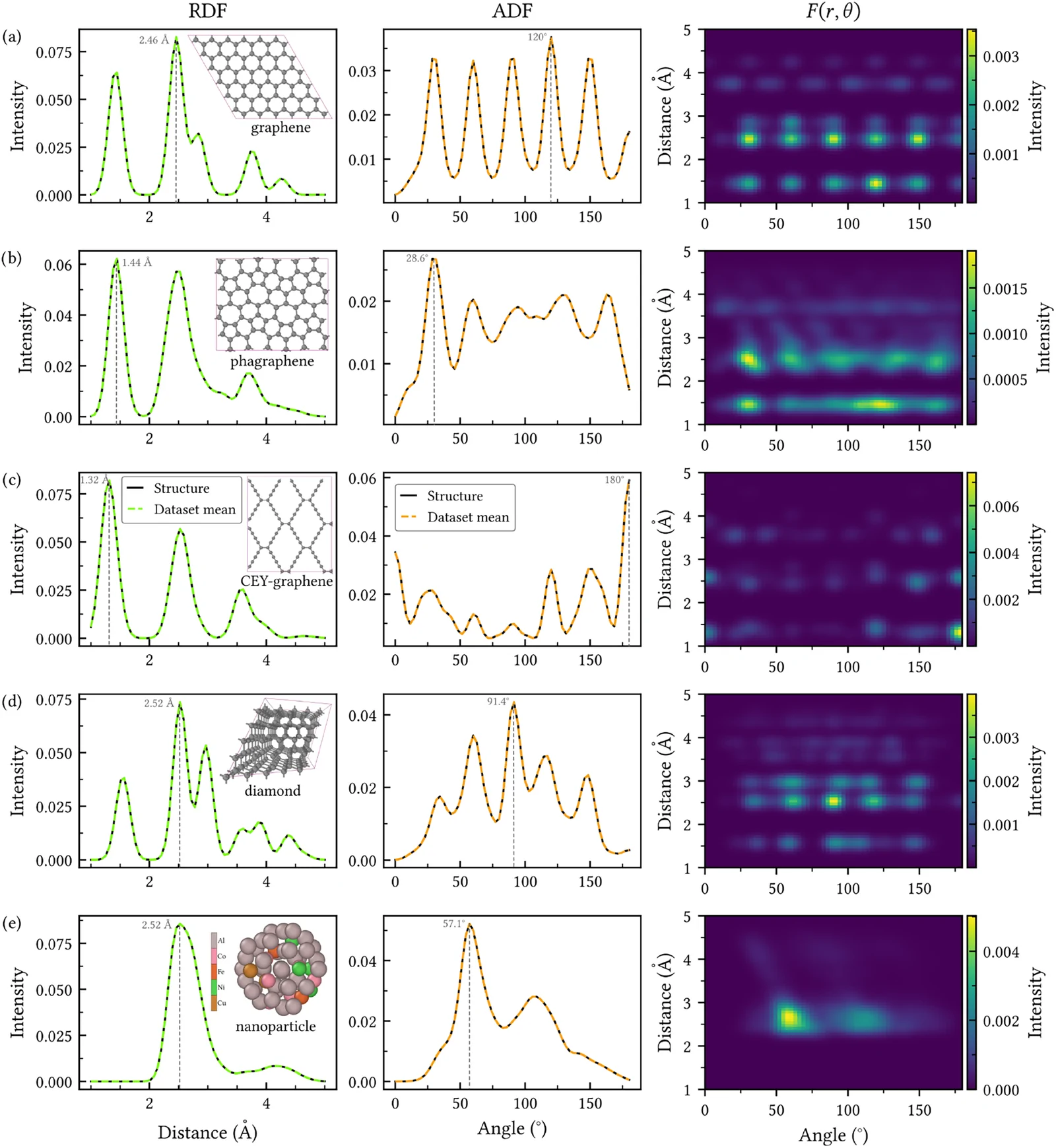
Structural fingerprints generated by the RACE descriptor for representative atomistic systems with different bonding topologies, dimensionalities, and chemical complexities. For each system, the radial distribution function (RDF), angular distribution function (ADF), and joint radial–angular correlation map *F*(*r*, θ) are shown. Numerical annotations and dashed guides identify representative characteristic RDF distances and key ADF angles; broad higher-shell features in the more disordered systems are left unlabeled to avoid assigning spurious precision to overlapping maxima. The panels correspond to (a) graphene, a prototypical two-dimensional sp^2^ carbon network; (b) phagraphene, a topologically complex carbon-allotrope containing nonhexagonal rings; (c) CEY-graphene, a planar carbon network with a distinct bonding architecture; (d) diamond, a three-dimensional sp^3^ covalent crystal; and (e) a finite multicomponent nanoparticle with stoichiometry Al_70_Co_10_Fe_5_Ni_10_Cu_5_.

Graphene, shown in Figure 1a, provides a reference example of an ordered 2D sp^2^ carbon network. Its RDF exhibits sharp coordination-shell peaks, with the first maximum near the characteristic C–C bond length of graphene, while the ADF is dominated by the trigonal planar angle around 120°. The corresponding joint map shows a localized intensity pattern associated with the coupling between short C–C bonds and the sp^2^ angular environment. This compact signature reflects the high structural regularity of the honeycomb lattice.

More complex planar carbon networks, such as phagraphene and CEY-graphene in Figure 1b and Figure 1c, yield broader, more structured descriptor profiles. Although their first-neighbor RDF peaks remain close to those expected for sp^2^-bonded carbon, the higher coordination shells and angular distributions are more diverse than in graphene. This behavior reflects the presence of nonhexagonal rings and multiple local bonding motifs. In the joint maps, the intensity is distributed over a wider region of radial and angular space, indicating that the structural complexity of these allotropes is encoded not only by bond lengths or bond angles separately, but also by their correlations.

The diamond structure in Figure 1d illustrates the sensitivity of RACE to changes in dimensionality and bonding hybridization. In contrast to the planar carbon systems, diamond exhibits a three-dimensional sp^3^ covalent network, with a dominant angular feature near the tetrahedral angle of 109.5°. The joint radial–angular map clearly separates this tetrahedral coordination motif from the trigonal planar signatures observed in the sp^2^ systems.

The finite multicomponent nanoparticle in Figure 1e represents a more heterogeneous case. Its RDF is broader than those of the crystalline carbon systems, reflecting the coexistence of multiple metallic bond lengths and coordination environments. The ADF also spans a wider angular range, consistent with the structural diversity expected in finite metallic clusters containing chemically distinct species and surface atoms with reduced coordination. The joint map preserves this information as a distributed intensity pattern, capturing the combined effects of chemical heterogeneity, finite-size disorder, and local packing motifs.

Importantly, Figure 1 demonstrates the component-level interpretability of RACE. Ordered structures produce sharp radial and angular signatures and localized joint maps, whereas topological, chemical, and finite-size disorder broaden these distributions in physically recognizable ways.

### Structurally Controlled Carbon Benchmarks

Figure 2 consolidates the carbon-allotrope, vacancy-defective-graphene, and distorted diamond benchmarks. RACE, conventional SOAP, ACSF, and ACE are compared using 20 common partitions, training-only feature selection, MAE, RMSE, and 95% confidence intervals.

**Figure 2:**
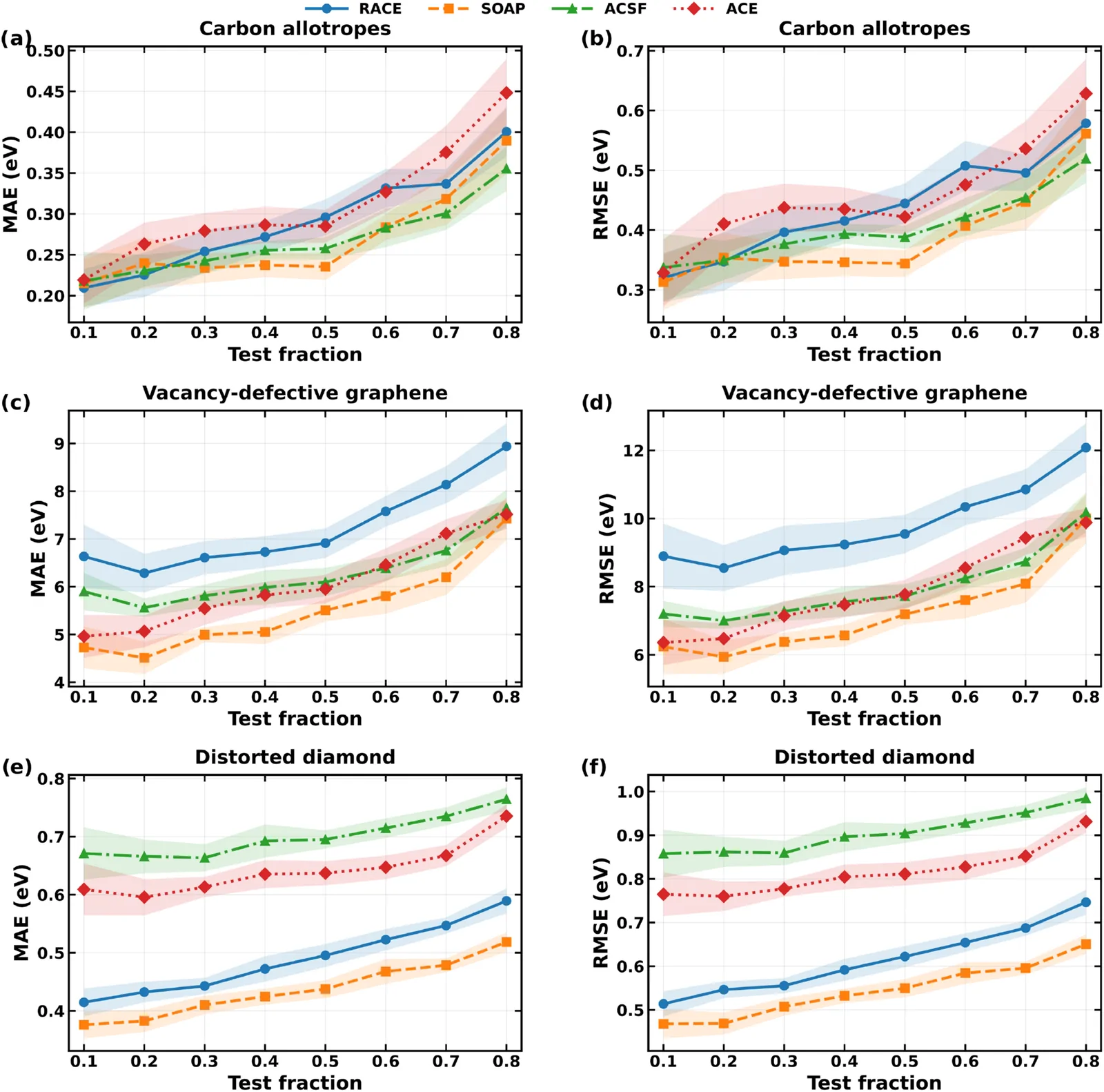
Predictive performance for structurally controlled carbon systems. The rows correspond to carbon allotropes, vacancy-defective graphene, and distorted diamond, and the columns report MAE and RMSE. Curves show the mean over 20 repeated partitions at each test fraction, and shaded regions denote 95% confidence intervals. All representations use identical split indices, and feature selection is fitted only on the corresponding training subset. The compared descriptors are RACE, conventional SOAP, ACSF, and ACE.

In the carbon-allotrope data set, the relative ordering changes with test fraction and metric. RACE remains within the same broad error regime as the alternatives, but SOAP and ACSF reach lower errors in several partitions. Overlapping confidence intervals at multiple fractions further show that small differences should not be interpreted as a general ranking.

For vacancy-defective graphene, conventional SOAP gives the lowest MAE and RMSE over most of the evaluated range, whereas RACE exhibits larger errors. The structure-level mean and standard deviation may dilute the localized vacancy environment within a large supercell. This result identifies a limitation of the present global pooling rather than evidence for universal descriptor inadequacy. Consequently, future work could investigate defect-centered or locality-sensitive aggregation.

For distorted diamond, SOAP again gives the lowest errors over most fractions. RACE performs better than ACSF and ACE in this benchmark but remains less accurate than SOAP. This outcome is consistent with SOAP’s sensitivity to smooth perturbations of a chemically homogeneous neighbor density. The distorted configurations were included precisely as a controlled test of such continuous geometric perturbations at fixed composition and topology, not as a case selected to favor RACE.

Figure 2 therefore establishes RACE as a useful, interpretable baseline rather than a uniformly leading predictor. Complete *R* ^2^ values and out-of-fold parity plots are supplied in the Supporting Information.

### Internal Ablation of the RACE Representation

Figure 3 tests RDF, ADF, RDF+ADF, the joint *F*(*r*, θ) block, and full RACE using native component dimensions and identical repeated 80/20 partitions.

**Figure 3:**
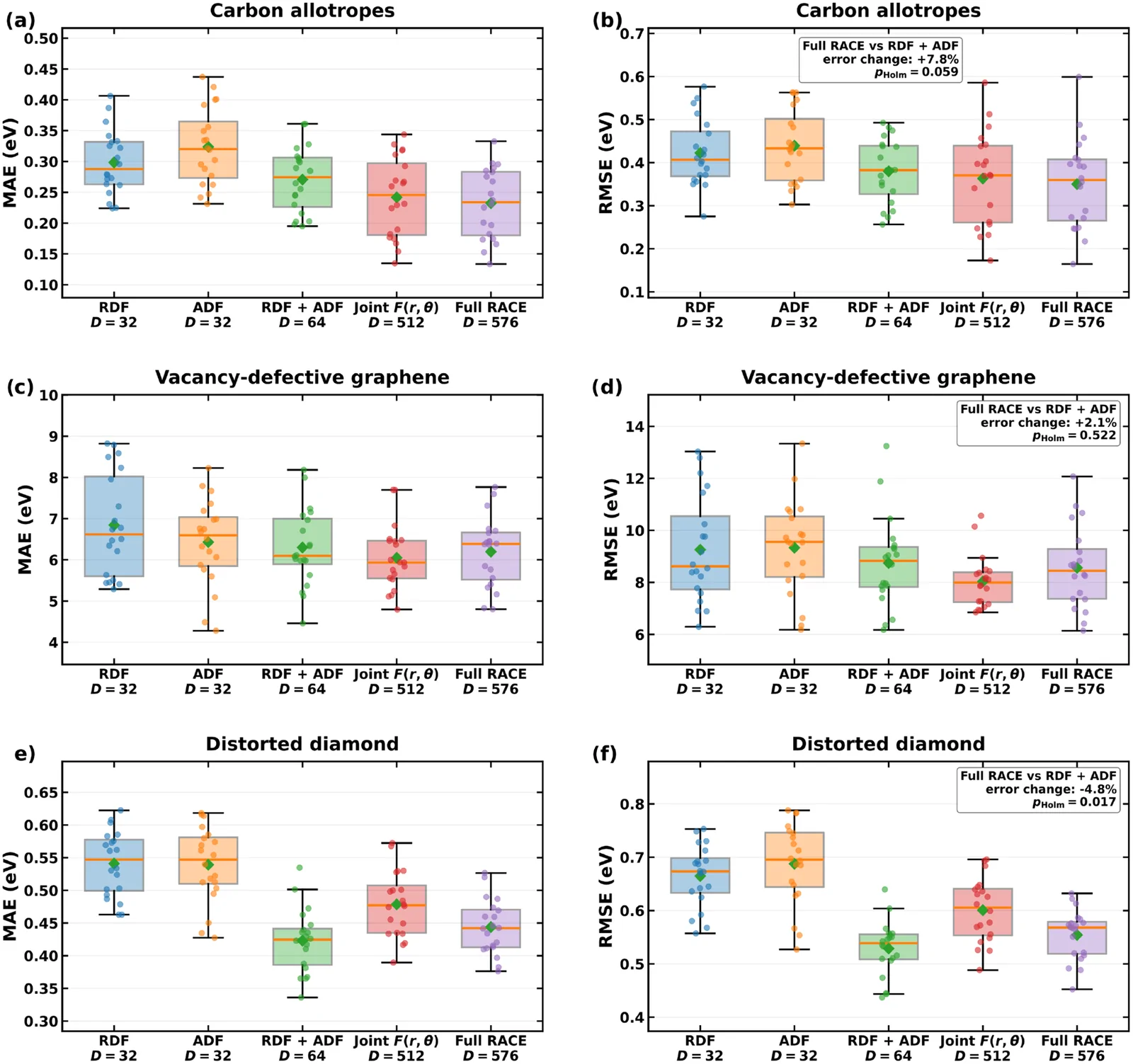
Internal ablation of RACE for the three carbon benchmarks. The representations are RDF (*D* = 32), ADF (*D* = 32), RDF+ADF (*D* = 64), joint *F*(*r*, θ) (*D* = 512), and full RACE (*D* = 576). Box boundaries denote the interquartile range, horizontal lines are medians, diamonds are means, and points are results from 20 repeated 80/20 partitions. Annotations compare full RACE with RDF+ADF using paired Wilcoxon tests with Holm correction. Positive reduction denotes lower RMSE for full RACE; negative reduction denotes increased RMSE.

The joint block is predictive on its own, but its incremental value after concatenation with RDF and ADF is system dependent. For carbon allotropes, full RACE reduces mean RMSE relative to RDF+ADF by approximately 7.8%, although the Holm-corrected value (*p* = 0.059) lies slightly above the conventional 0.05 threshold. For vacancy-defective graphene, the reduction is approximately 2.1% and is not significant (*p* = 0.522). For distorted diamond, full RACE increases RMSE by approximately 4.8% relative to RDF+ADF, with a significant paired difference (*p* = 0.017).

The joint map therefore contains structural information but is not a universally beneficial addition. In finite-data regimes, its larger dimension can introduce redundancy or variance. Full RACE should be viewed as one configurable member of a descriptor family rather than as a mandatory optimum. This analysis also demonstrates why feature-importance rankings alone cannot establish component necessity. The selected features are reported in the Supporting Information.

### Chemically Complex Systems

Figure 4 presents a consolidated comparison of RACE, conventional SOAP, compressed SOAP-μ_2_, ACSF, and ACE across high-entropy alloy, nanoparticle, and Materials Project benchmarks. The comparison evaluates both MAE and RMSE using consistent data partitions and training-only feature selection.

**Figure 4:**
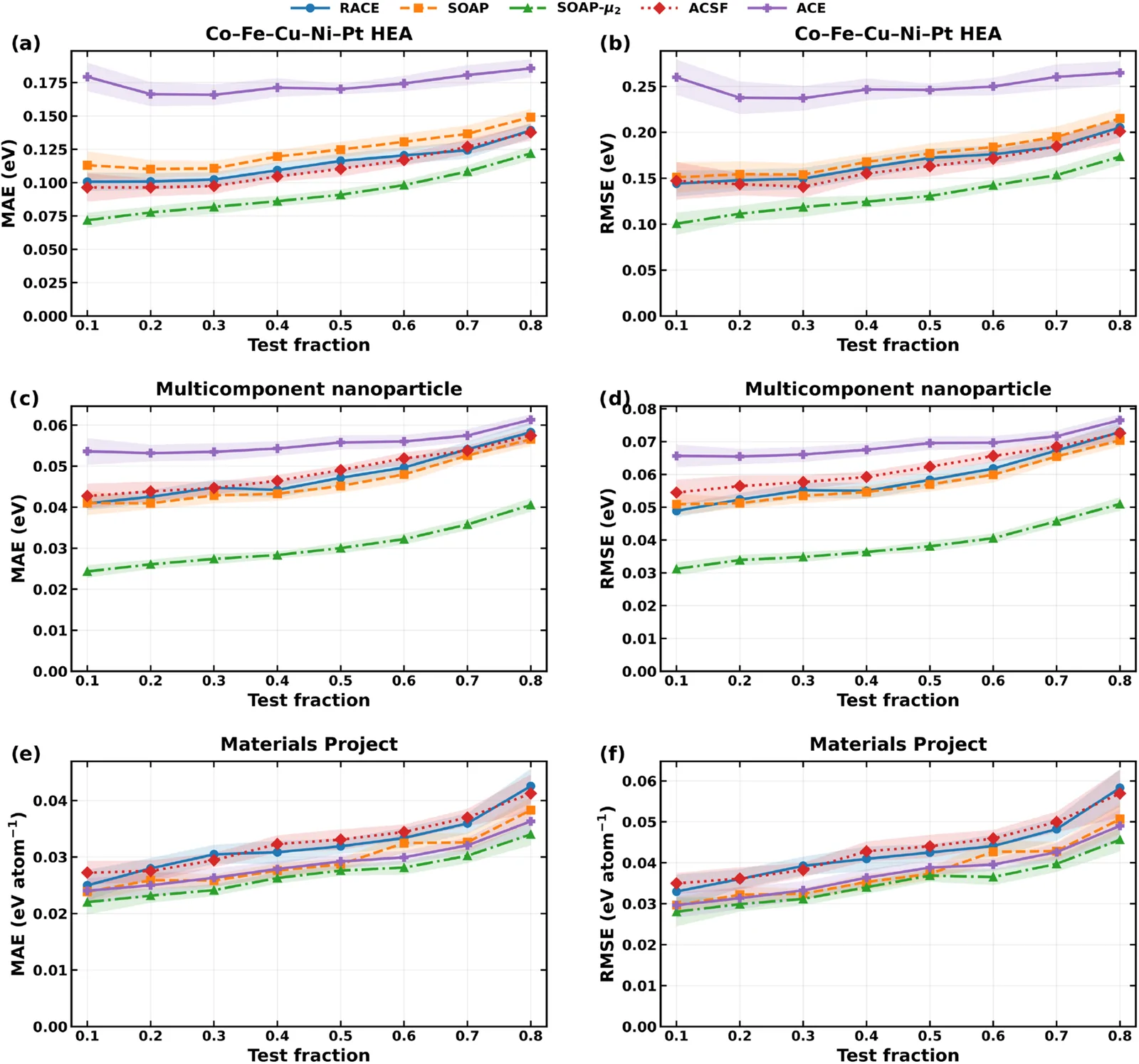
Descriptor comparison for chemically complex systems. The rows correspond to the Co–Fe–Cu–Ni–Pt high-entropy alloy, the multicomponent nanoparticle, and the Materials Project subset, whereas the columns show MAE and RMSE. Curves are means over 20 repeated partitions and shaded regions denote 95% confidence intervals. All methods use common split indices and training-only feature selection. Materials Project errors are reported in eV/atom.

SOAP-μ_2_ gives the lowest errors across the three chemically complex benchmarks. This result shows that compressed SOAP substantially controls chemical-channel growth while retaining strong predictive performance. RACE remains operational and useful over the same chemical spaces, but the data do not support a claim of superior chemical accuracy.

For the high-entropy alloy, RACE, conventional SOAP, and ACSF occupy a relatively close performance range, while SOAP-μ_2_ is consistently more accurate and the tested ACE representation has larger errors. For the nanoparticle, SOAP-μ_2_ again provides a clear error reduction. Conventional SOAP and RACE are similar, with minor ordering changes as the training fraction varies. For Materials Project, SOAP-μ_2_ and ACE are favorable, whereas differences among the remaining fixed representations are modest at several fractions.

The contribution of RACE in multicomponent systems is therefore not unique feasibility. Its distinguishing property is a fixed-dimensional scalar chemical map combined with directly inspectable radial, angular, and joint channels. Compressed SOAP provides a stronger predictive baseline in these data sets, while RACE provides a simpler component-level decomposition.

### Sensitivity to Chemical Encoding

Figure 5 compares geometry-only RACE, atomic-number weighting, electronegativity, covalent radius, valence-electron count, and a multichannel representation.

**Figure 5:**
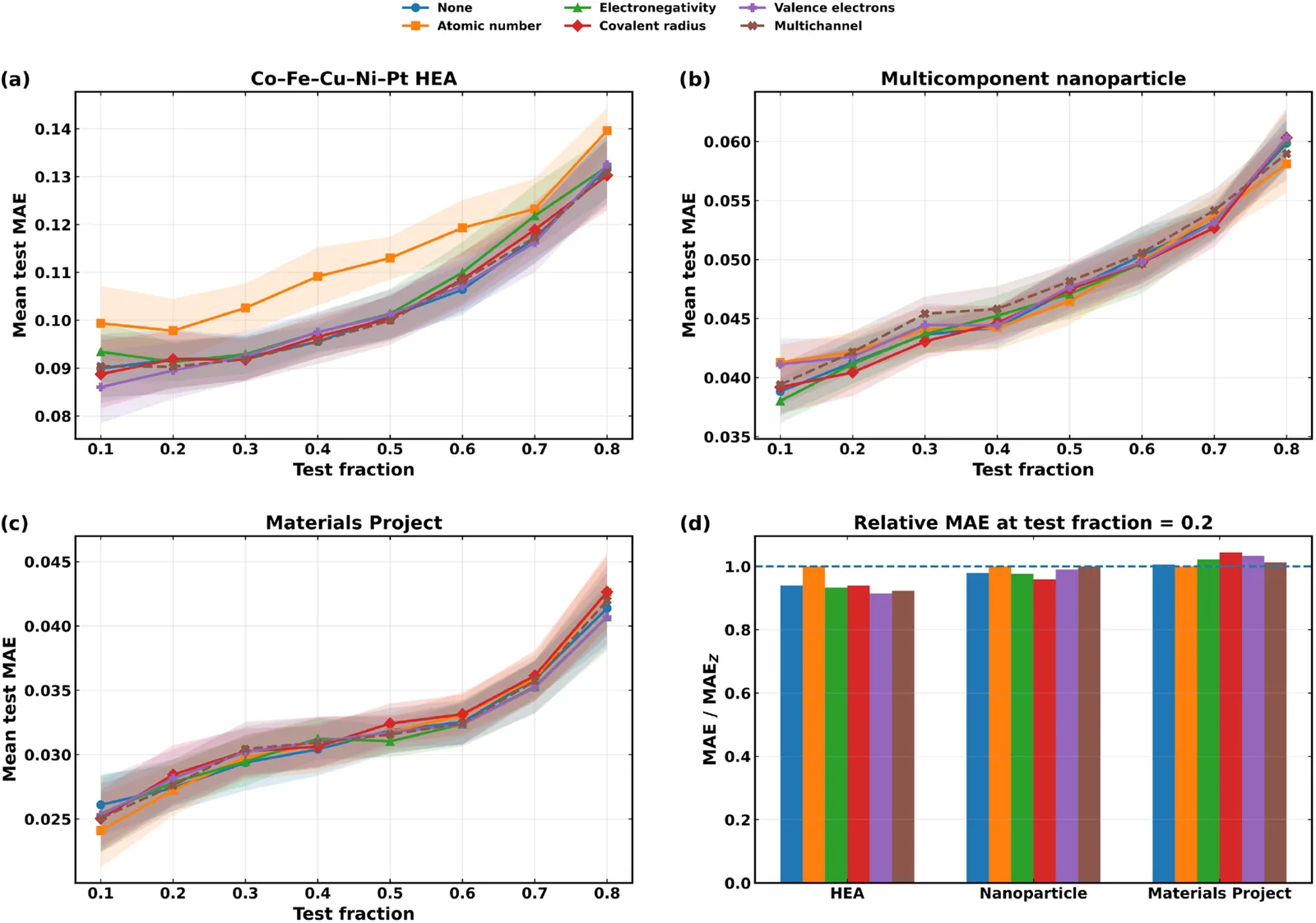
Sensitivity of RACE to chemical encoding. Panels (a)–(c) show mean test MAE as a function of test fraction for the HEA, nanoparticle, and Materials Project data sets. Panel (d) reports MAE at test fraction 0.2 normalized by the atomic-number result for each data set. Values below unity indicate lower error than the atomic-number baseline. HEA and nanoparticle errors are in eV, whereas Materials Project errors are in eV/atom. Shaded regions denote 95% confidence intervals across repeated partitions.

No scalar map is optimal across all systems. In the HEA, several alternatives improve on atomic-number weighting, with valence-electron and multichannel representations among the lower error variants at small and intermediate test fractions. For the nanoparticle, the curves are close and their uncertainty bands overlap substantially. For Materials Project, the differences are again small and atomic number is not consistently favored.

Atomic number is therefore retained only as a simple, universally available default. It is not a metric of chemical similarity. Different elemental properties encode different aspects of bonding, and their utility depends on the data set and target. For the Co–Fe–Cu–Ni–Pt HEA in particular, none of these scalar maps represents spin configuration or local magnetic moments, so the differences in Figure 5 should not be interpreted as a test of magnetic-state resolution. Magnetic-state-aware applications would require an explicit magnetic degree of freedom in the representation or model. The multichannel option increases flexibility but also increases dimension and may introduce redundant correlations. Learned low-rank chemical maps constitute a natural future extension.

### Materials Project Generalization and the Role of Composition

Figure 6 compares random partitions, reduced-formula-grouped partitions, and chemical-system-grouped partitions. The evaluated models are composition-only, RACE, RACE+composition, SOAP-μ_2_, and ACE.

**Figure 6:**
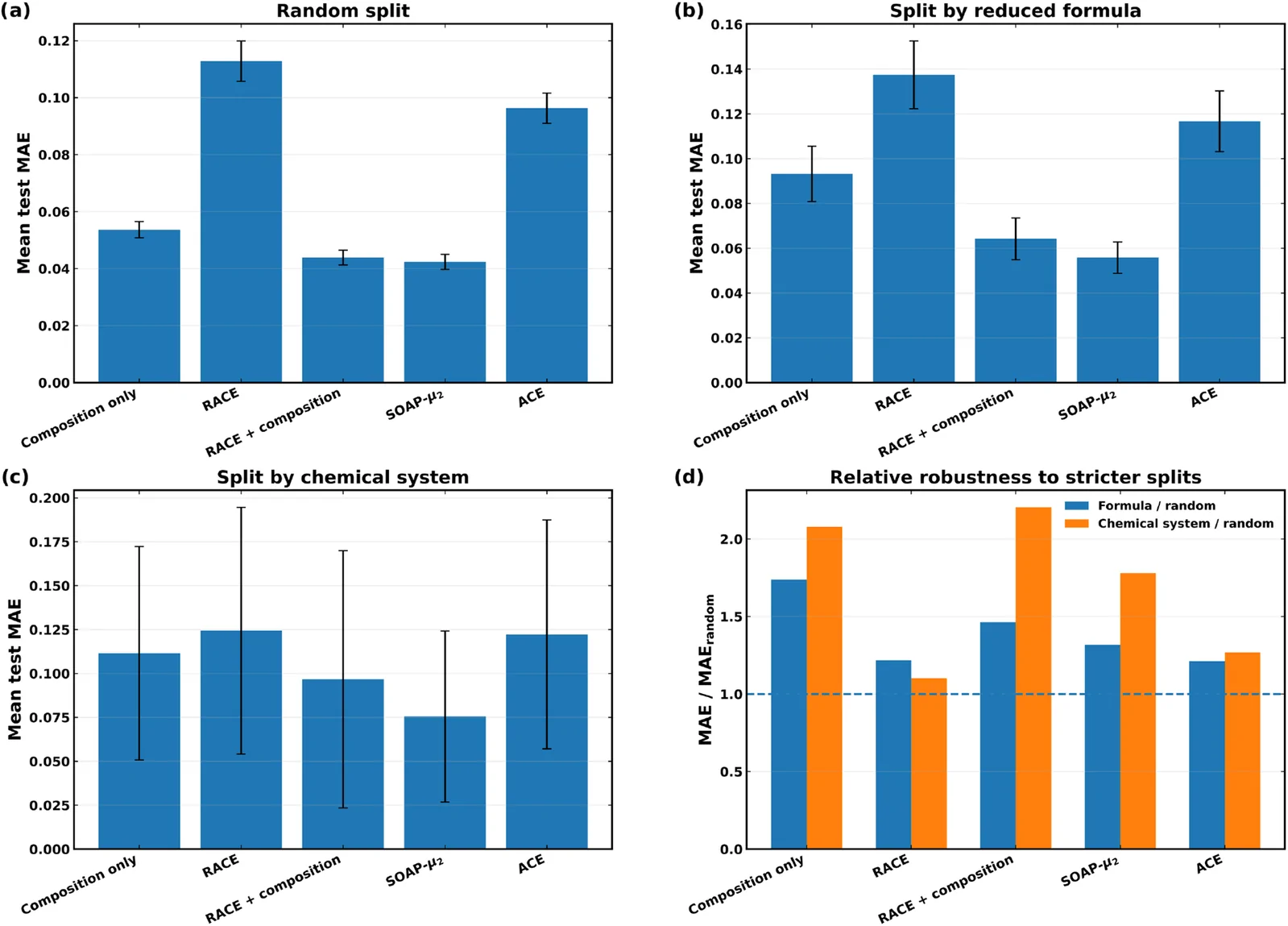
Robust Materials Project evaluation. Panels (a)–(c) report mean test MAE for random splits, splits grouped by reduced formula, and splits grouped by chemical-system. Panel (d) reports grouped-split MAE divided by the corresponding random-split MAE. Values above unity indicate degradation under a stricter extrapolative partition. Errors are in eV/atom. Grouped splits are evaluated only when at least two distinct groups exist, whereas small group sets use leave-one-group-out validation.

The random-split analysis shows that standalone RACE does not adequately represent the full chemical variation of this database. Its MAE is larger than the composition-only baseline, whereas RACE+composition reduces the error to the range of SOAP-μ_2_. Scalar chemical weighting within a local descriptor therefore does not replace an explicit representation of global stoichiometry.

All methods deteriorate under formula- and chemical-system-grouped validation, but the magnitude of deterioration differs. SOAP-μ_2_ provides the lowest absolute MAE under the grouped protocols in this benchmark. RACE alone remains comparatively weak, whereas RACE+composition is substantially improved. The composition-only baseline also degrades strongly for unseen chemical systems, demonstrating that stoichiometry without local structure is not sufficient.

RACE should consequently be interpreted as a local structural representation with a compact chemical map. For broad heterogeneous databases, it is more appropriately combined with explicit composition or embedded in an architecture that treats local geometry and global stoichiometry separately.

### Descriptor Dimensionality and Computational Characteristics

Figure 7 summarizes the native dimension, storage, peak memory, descriptor generation time, training time, species scaling, and atom-count scaling of the evaluated descriptors.

**Figure 7:**
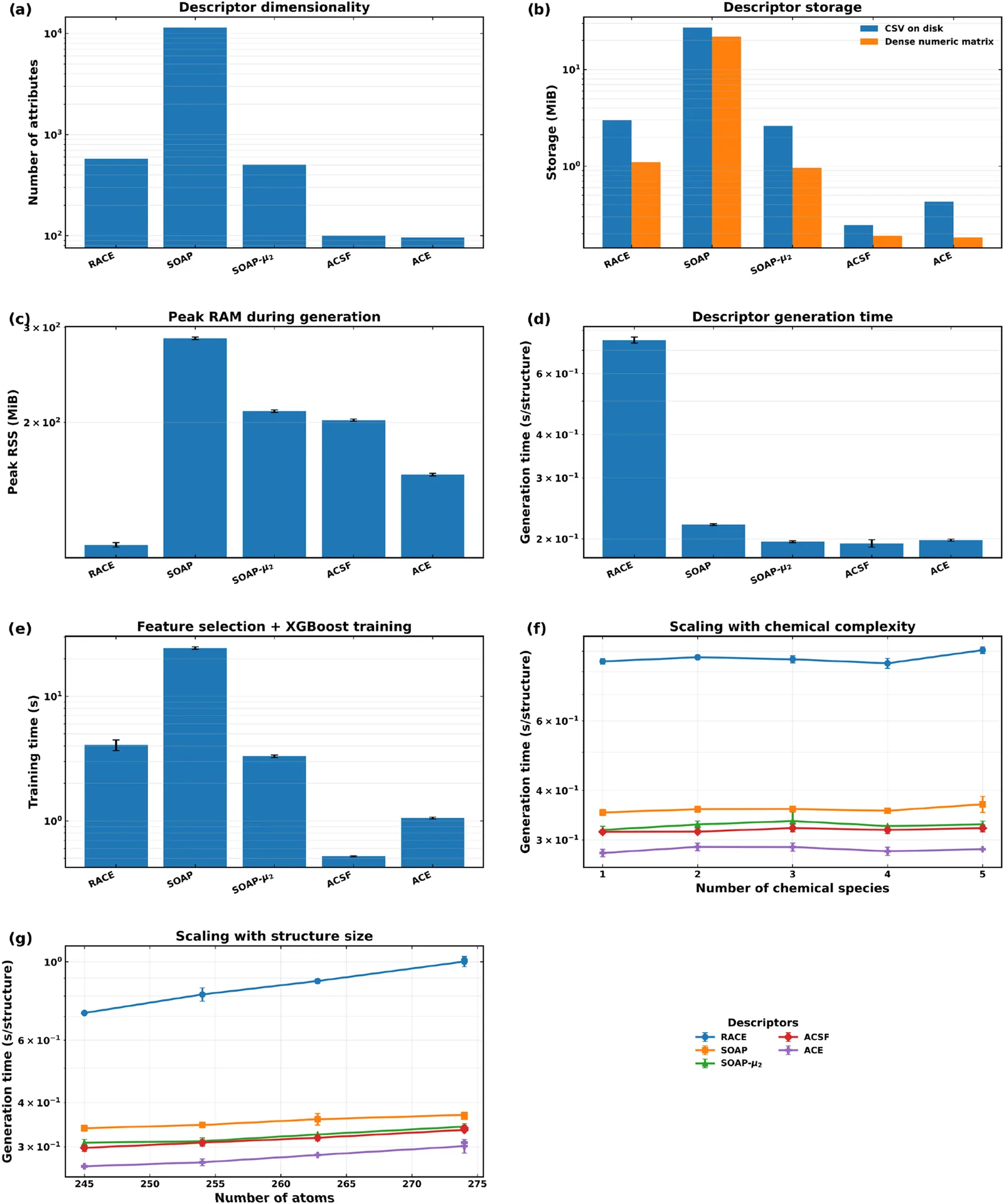
Computational characteristics of the evaluated descriptors. (a) Native descriptor dimensionality. (b) Storage requirements for CSV and dense-matrix representations. (c) Peak resident memory during descriptor generation. (d) Descriptor-generation time per structure. (e) Feature selection and XGBoost training time. (f) Descriptor-generation time as a function of the number of chemical species at comparable atom counts. (g) Descriptor-generation time as a function of the number of atoms for pure-carbon structures. Generation benchmarks were performed using a fresh process and a single CPU thread, with peak RAM corresponding to the maximum resident set size of the process tree. Error bars denote 95% confidence intervals from repeated measurements.

Conventional SOAP has by far the largest native dimension and storage requirement in the representative multicomponent benchmark. SOAP-μ_2_ reduces the dimension to a value comparable to RACE, while ACSF and the tested ACE representation are smaller. RACE is therefore compact relative to conventional SOAP but is not the smallest representation considered.

RACE exhibits the lowest peak resident memory during descriptor generation. This is practically relevant for memory-constrained or sequential workflows. However, it is the slowest generator among the tested implementations because the explicit angular and joint channels require neighbor-pair operations. Low peak memory must therefore not be conflated with low wall-clock cost.

Conventional SOAP is also the most expensive representation during feature selection and model training because of its large native matrix. Compressed SOAP, RACE, ACSF, and ACE are substantially cheaper at this stage. RACE thus combines low generation memory with moderate downstream training cost, but it is not generation time optimal.

The species scaling analysis clarifies the appropriate scalability claim. The single-channel RACE dimension is independent of the number of species, and its generation time changes only weakly across the tested chemical alphabet. Compressed SOAP provides a similarly effective control of chemical dimensionality. We therefore use the precise statement ”species-independent descriptor dimension” instead of claiming universal computational scalability.

## Limitations and Scope

RACE is not a complete invariant basis, and its finite grid may omit information retained by systematic density expansions. The joint channel can add redundancy and is not uniformly beneficial. Scalar chemical maps do not fully represent global composition or subtle chemical similarity. Mean-and-standard-deviation pooling can dilute localized defects. Finally, the current Python implementation is slower than the alternative descriptor generators evaluated here.

The study also does not establish a general ranking relative to modern learned equivariant representations. Such models optimize task-specific geometric and chemical features and normally require a distinct architecture, training protocol, and hyperparameter search. A scientifically controlled comparison would require larger data sets and is beyond the fixed-descriptor protocol used here. No claim of superiority over learned representations is made.

## Conclusions

The Radial-Angular Correlation Descriptor (RACE) is introduced as a modular radial–angular representation composed of explicit RDF, ADF, and optional joint *F*(*r*, θ) channels. Chemical identity enters through interchangeable scalar or multichannel maps, allowing the geometric representation and chemical encoding to be modified independently.

We checked that RACE provides useful predictive accuracy but does not uniformly outperform conventional SOAP, compressed SOAP, ACSF, or ACE. SOAP performs particularly well for vacancy-defective graphene and distorted diamond, while SOAP-μ_2_ gives the lowest errors in the chemically complex benchmarks. The ablation demonstrates that the joint radial–angular channel contains predictive information but is not universally beneficial when concatenated with RDF and ADF. Atomic-number weighting is similarly useful as a simple default but is not consistently optimal.

The Materials Project analysis demonstrates that a local structural representation with scalar chemical weighting is not a substitute for explicit global composition. Combining RACE with elemental fractions substantially improves performance, while grouped partitions quantify the loss of accuracy associated with extrapolation to unseen formulas and chemical systems.

The computational results also require a qualified interpretation. RACE has a species-independent feature dimension in its scalar-weighted form and low peak memory during descriptor generation. It is compact relative to conventional SOAP and less expensive than conventional SOAP during downstream feature selection and fitting. Its present implementation is nevertheless not the fastest descriptor generator, and compressed SOAP, ACSF, or ACE may be smaller or more accurate depending on the application.

RACE should therefore be viewed neither as a universal replacement for SOAP nor as a new systematic many-body theory. Its contribution is a transparent and configurable organization in which radial, angular, joint, and chemical channels can be inspected and manipulated directly. This makes it useful as a physically interpretable baseline, a platform for descriptor ablation, and a component that can be combined with explicit composition or other global information. Future work will address optimized implementations, adaptive selection of joint features, learned low-rank chemical maps, defect-centered pooling, and integration with equivariant or graph-based models.

## Supplementary Material



## Data Availability

The Python implementation of the RACE descriptor is publicly available on GitHub at https://github.com/tromer-unb/RACE.

## Notes

Out-of-fold parity plots for the principal benchmark systems; distributions of MAE, RMSE, and *R* ^2^ over the ten repeated out-of-fold validation runs; repeat-wise out-of-fold performance and paired MAE comparisons; most stable selected feature for every complex-system/descriptor combination at a 20% test fraction (PDF)
