# Supplementary material for: RACE: A Modular and Interpretable Radial–Angular Correlation Descriptor for Atomistic Machine Learning
Source: J Chem Theory Comput. 2026 Oct 8;22(20):10914–27. doi: 10.1021/acs.jctc.6c01111 (PMC13718549; doi:10.1021/acs.jctc.6c01111)
Supplement: Supplementary file 1 [file ct6c01111_si_001.pdf]

Supporting Information for

RACE: A Modular and Interpretable Radial–Angular Correlation Descriptor for Atomistic Machine Learning

Raphael M. Tromer,[†] Jhionathan de Lima,[‡,¶] Cristiano Francisco Woellner,[‡,¶] Paola Ferreira Barbosa,[§] Roberto Ventura Santos,[§] and Luiz Antonio Ribeiro Junior[*,‖,#]

[†]*University of Brasília, Institute of Physics, Brasília, Federal District, 70910-900, Brazil*

[‡]*Department of Physics, Federal University of Paraná, 81531-980, Curitiba, PR, Brazil*

[¶]*Interdisciplinary Center for Science, Technology, and Innovation (CICTI), Federal University of Paraná, 81530-000, Curitiba, PR, Brazil*

[§]*Institute of Geosciences, University of Brasília, DF, Brasília, 70910-900, Brazil*

[‖]*Department of Physics, Norwegian University of Science and Technology (NTNU), NO-7491 Trondheim, Norway*

[#]*Computational Materials Laboratory (LCCMat), Institute of Physics, University of Brasília, 70910-900, Brasília, Federal District, Brazil*

E-mail: luiz.a.ribeiro@ntnu.no

This Supporting Information provides prediction-level diagnostics and compact summaries of the repeated validation, feature selection, descriptor ablation, chemical encoding, generalization, and scalability analyses. Complete seed-level and partition-level data remain available in the machine-readable CSV files from which the tables below were derived.

Out-of-fold prediction diagnostics

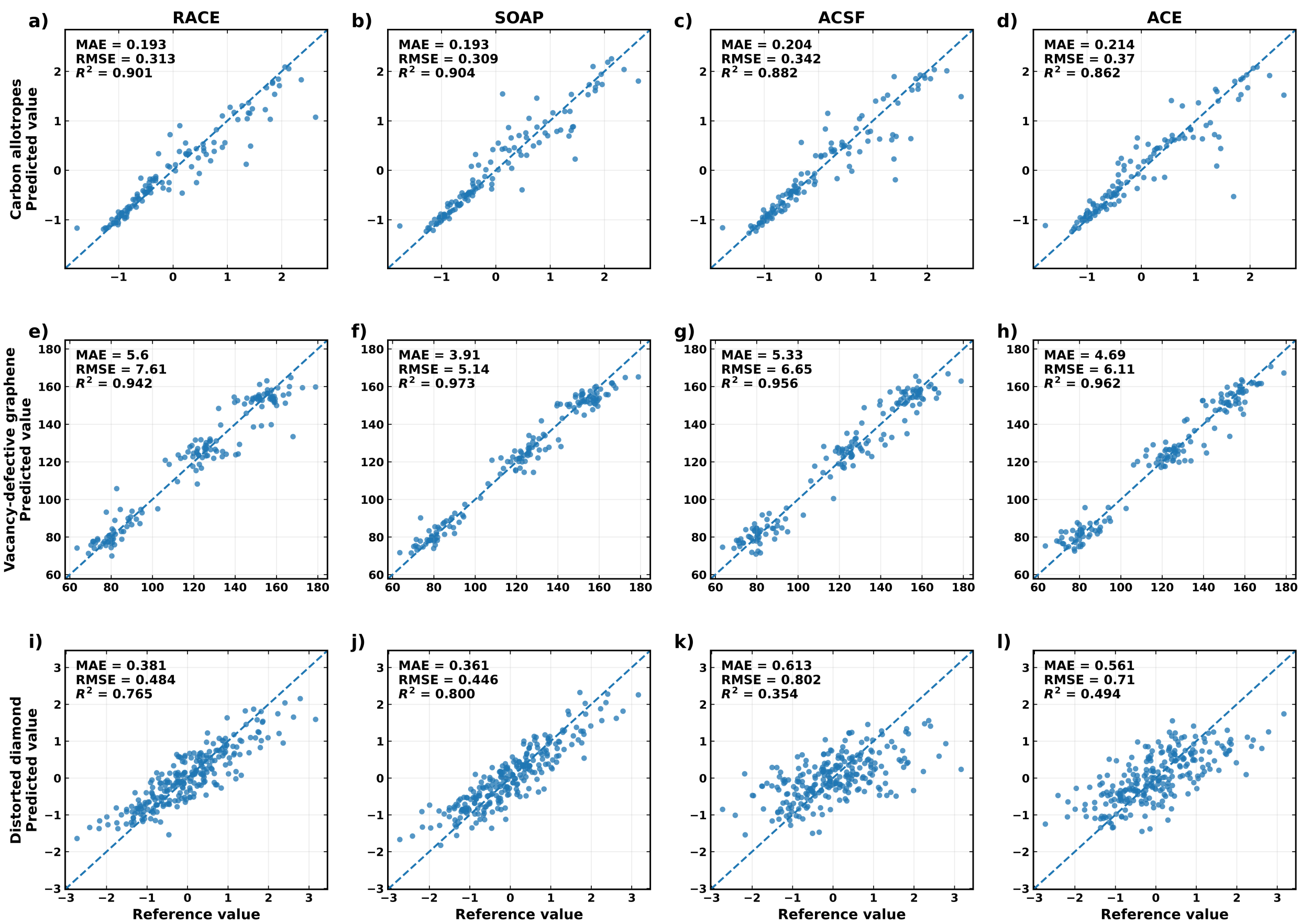

Fig. S1: Out-of-fold parity plots for the principal benchmark systems. Columns correspond to RACE, SOAP, ACSF, and ACE, and rows correspond to carbon allotropes, vacancy-defective graphene, and distorted diamond. Each point is the prediction averaged over repeated out-of-fold cross-validation, and the dashed line denotes ideal agreement. The panel annotations report the metrics of the averaged out-of-fold predictions.

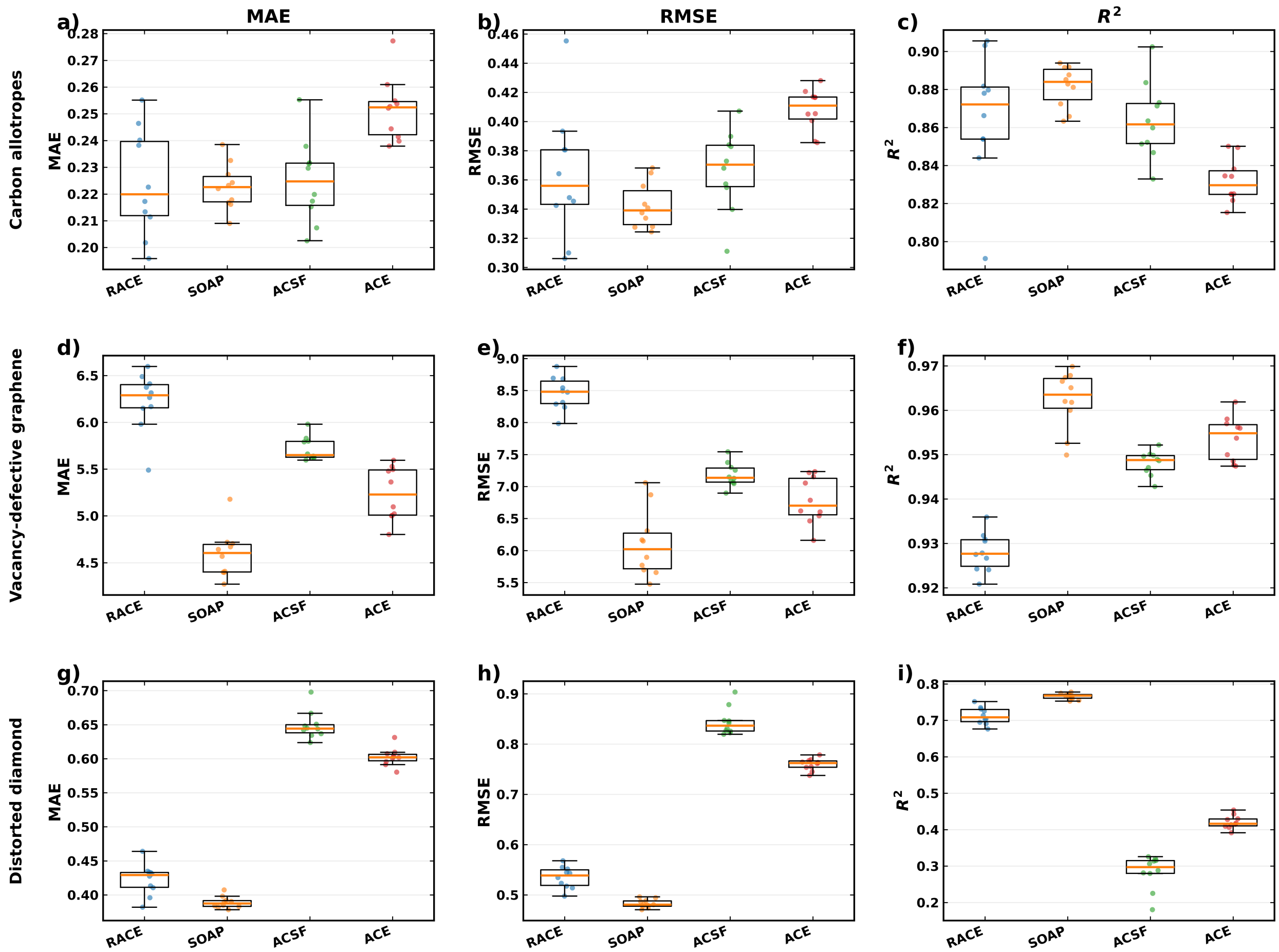

Fig. S2: Distributions of MAE, RMSE, and R^2 over the ten repeated out-of-fold validation runs. Points show individual repeats and boxes summarize their distributions. SOAP gave the lowest repeat-wise MAE for vacancy-defective graphene and distorted diamond, whereas the differences among RACE, SOAP, and ACSF were small for the carbon allotrope benchmark.

Principal predictive performance

Table S1: Repeat-wise out-of-fold performance and paired MAE comparisons. Values are mean ± standard deviation over ten repeats. ΔMAE is the descriptor MAE minus the RACE MAE for the same system; negative values favor the alternative descriptor. "Wins" is the number of paired repeats with lower MAE than RACE. The final column gives the Holm-adjusted two-sided Wilcoxon p-value. The lowest mean MAE in each system is bold.

System	Descriptor	MAE	RMSE	R^2	ΔMAE vs RACE	Wins/10	p_{Holm}
Carbon allotropes	RACE	0.224 ± 0.020	0.363 ± 0.044	0.866 ± 0.033	–	–	–
	SOAP	**0.223 ± 0.009**	**0.342 ± 0.016**	**0.882 ± 0.011**	-0.001	6/10	1.0000
	ACSF	0.225 ± 0.016	0.367 ± 0.028	0.864 ± 0.020	+0.001	5/10	1.0000
	ACE	0.252 ± 0.012	0.408 ± 0.014	0.832 ± 0.012	+0.027	2/10	0.0391
Vacancy-defective graphene	RACE	6.224 ± 0.314	8.458 ± 0.260	0.928 ± 0.004	–	–	–
	SOAP	**4.595 ± 0.257**	**6.105 ± 0.523**	**0.962 ± 0.007**	-1.629	10/10	0.0117
	ACSF	5.718 ± 0.126	7.183 ± 0.187	0.948 ± 0.003	-0.506	9/10	0.0117
	ACE	5.239 ± 0.283	6.783 ± 0.367	0.954 ± 0.005	-0.985	10/10	0.0117
Distorted diamond	RACE	0.422 ± 0.023	0.535 ± 0.022	0.712 ± 0.023	–	–	–
	SOAP	**0.389 ± 0.009**	**0.483 ± 0.008**	**0.766 ± 0.008**	-0.033	9/10	0.0117
	ACSF	0.649 ± 0.021	0.844 ± 0.027	0.284 ± 0.047	+0.227	0/10	0.0117
	ACE	0.602 ± 0.013	0.759 ± 0.012	0.421 ± 0.018	+0.180	0/10	0.0117

The repeat-wise comparison shows that RACE and SOAP were statistically indistinguishable for carbon allotropes, while RACE outperformed ACE. SOAP significantly improved the vacancy-defective graphene and distorted diamond MAEs relative to RACE. Across the separate learning-curve analysis, SOAP had the lowest MAE for vacancy-defective graphene and distorted diamond at every evaluated test fraction (0.1–0.8). For carbon allotropes, the lowest MAE shifted from RACE at test fractions 0.1–0.2, to SOAP at 0.3–0.5, and to ACSF at 0.6–0.8.

Feature-selection stability and descriptor dimensionality

Table S2: Most stable selected feature for every complex-system/descriptor combination at a 20% test fraction. The native dimension is the number of descriptor columns before selection. Selection frequency is the fraction of 20 repeated train/test partitions in which the feature was retained.

System	Descriptor	Native dim.	Top feature	Selected runs	Frequency	Mean rank	Best rank
Co–Fe–Cu–Ni–Pt HEA	RACE	576	`adf_mean_02`	20/20	1.00	11.55	7
	SOAP	11480	`soap_09054`	14/20	0.70	2.64	1
	SOAP-μ_2	504	`soap_mu2_000369`	20/20	1.00	1.40	1
	ACSF	100	`acsf_0067`	20/20	1.00	1.30	1
	ACE	222	`ace_00049`	19/20	0.95	5.32	1
Multicomponent nanoparticle	RACE	576	`joint_mean_0102`	20/20	1.00	7.15	1
	SOAP	11480	`soap_00245`	20/20	1.00	2.00	1
	SOAP-μ_2	504	`soap_mu2_000030`	20/20	1.00	1.10	1
	ACSF	100	`acsf_0000`	20/20	1.00	1.00	1
	ACE	222	`ace_00002`	20/20	1.00	7.10	1
Materials Project	RACE	576	`joint_mean_0035`	20/20	1.00	24.35	14
	SOAP	45360	`soap_00000`	20/20	1.00	4.65	2
	SOAP-μ_2	504	`soap_mu2_000000`	20/20	1.00	5.55	3
	ACSF	300	`acsf_0004`	20/20	1.00	1.55	1
	ACE	96	`ace_00000`	20/20	1.00	4.10	2

For RACE, the most persistent variables were an angular-distribution feature for the high-entropy alloy and joint radial–angular features for the nanoparticle and Materials Project datasets. SOAP-μ_2, ACSF, and several ACE features were selected in nearly every repeat, whereas the highest-frequency conventional SOAP feature for the high-entropy alloy appeared in 70% of repeats.

RACE component ablation

Table S3: RACE ablation results. Values are mean ± standard deviation over 20 repeated partitions. ΔMAE is the reduced-variant MAE minus the Full RACE MAE. The adjusted p-value compares each reduced variant with Full RACE using paired Wilcoxon tests. The lowest mean MAE in each system is bold.

System	Variant	Features	MAE	RMSE	R^2	ΔMAE vs Full	p_{Holm}
Carbon allotropes	RDF	32	0.298 ± 0.052	0.422 ± 0.078	0.792 ± 0.143	+0.066	1.1×10^{-5}
	ADF	32	0.324 ± 0.062	0.439 ± 0.084	0.794 ± 0.070	+0.091	7.6×10^{-6}
	RDF + ADF	64	0.271 ± 0.053	0.380 ± 0.074	0.839 ± 0.078	+0.038	0.0014
	Joint $F(r,\theta)$	512	0.242 ± 0.065	0.363 ± 0.108	0.851 ± 0.084	+0.010	0.0192
	Full RACE	576	**0.232 ± 0.057**	**0.350 ± 0.106**	**0.864 ± 0.071**	–	–
Vacancy-defective graphene	RDF	32	6.844 ± 1.244	9.256 ± 2.051	0.911 ± 0.038	+0.647	0.2549
	ADF	32	6.430 ± 1.046	9.331 ± 1.839	0.911 ± 0.032	+0.233	0.8828
	RDF + ADF	64	6.299 ± 0.946	8.746 ± 1.746	0.922 ± 0.030	+0.102	0.8828
	Joint $F(r,\theta)$	512	**6.049 ± 0.782**	**8.033 ± 1.003**	**0.935 ± 0.018**	-0.148	0.8828
	Full RACE	576	6.197 ± 0.849	8.561 ± 1.584	0.925 ± 0.026	–	–
Distorted diamond	RDF	32	0.541 ± 0.050	0.665 ± 0.058	0.524 ± 0.118	+0.097	7.6×10^{-6}
	ADF	32	0.539 ± 0.058	0.688 ± 0.077	0.492 ± 0.146	+0.095	7.6×10^{-6}
	RDF + ADF	64	**0.423 ± 0.048**	**0.529 ± 0.051**	**0.700 ± 0.073**	-0.021	0.0306
	Joint $F(r,\theta)$	512	0.479 ± 0.050	0.601 ± 0.060	0.612 ± 0.094	+0.035	0.0306
	Full RACE	576	0.444 ± 0.042	0.554 ± 0.047	0.672 ± 0.072	–	–

Full RACE significantly improved over every reduced representation for the carbon allotrope benchmark. For vacancy-defective graphene, the joint radial–angular term alone had a slightly lower mean MAE than Full RACE, but the paired difference was not significant. For distorted diamond, RDF+ADF gave a lower mean MAE than Full RACE and the paired difference remained significant after Holm correction.

Chemical encoding

Table S4: Chemical-encoding results at a 20% test fraction. Values are mean ± standard deviation over 20 repeated partitions. ΔMAE is relative to atomic-number weighting. The adjusted p-value is from the paired Wilcoxon comparison with atomic-number weighting. The lowest mean MAE in each system is bold.

System	Encoding	Features	MAE	RMSE	R^2	ΔMAE vs Z	p_{Holm}
Co–Fe–Cu–Ni–Pt HEA	Unweighted	576	0.092 ± 0.014	0.134 ± 0.031	0.660 ± 0.099	-0.0059	0.0223
	Atomic number	576	0.098 ± 0.015	0.142 ± 0.025	0.604 ± 0.150	–	–
	Electronegativity	576	0.091 ± 0.009	0.130 ± 0.018	0.671 ± 0.088	-0.0065	0.0583
	Covalent radius	576	0.092 ± 0.013	0.133 ± 0.029	0.664 ± 0.094	-0.0059	0.0158
	Valence electrons	576	**0.090 ± 0.013**	**0.129 ± 0.031**	0.680 ± 0.115	-0.0083	0.0249
	Multichannel	2880	0.090 ± 0.013	0.130 ± 0.028	0.681 ± 0.092	-0.0075	0.0385
Multicomponent nanoparticle	Unweighted	576	0.041 ± 0.004	0.051 ± 0.005	0.692 ± 0.063	-0.0009	0.8098
	Atomic number	576	0.042 ± 0.004	0.052 ± 0.005	0.678 ± 0.071	–	–
	Electronegativity	576	0.041 ± 0.005	0.051 ± 0.006	0.693 ± 0.063	-0.0010	0.8098
	Covalent radius	576	**0.040 ± 0.005**	**0.050 ± 0.005**	**0.704 ± 0.059**	-0.0017	0.1812
	Valence electrons	576	0.042 ± 0.003	0.051 ± 0.005	0.695 ± 0.081	-0.0004	1.0000
	Multichannel	2880	0.042 ± 0.004	0.052 ± 0.004	0.678 ± 0.062	-0.0000	1.0000
Materials Project	Unweighted	576	0.027 ± 0.004	0.035 ± 0.006	0.936 ± 0.023	+0.0002	1.0000
	Atomic number	576	**0.027 ± 0.005**	**0.035 ± 0.006**	**0.937 ± 0.025**	–	–
	Electronegativity	576	0.028 ± 0.004	0.036 ± 0.006	0.935 ± 0.022	+0.0006	1.0000
	Covalent radius	576	0.028 ± 0.005	0.036 ± 0.007	0.931 ± 0.031	+0.0012	1.0000
	Valence electrons	576	0.028 ± 0.005	0.037 ± 0.007	0.930 ± 0.030	+0.0009	1.0000
	Multichannel	2880	0.028 ± 0.005	0.035 ± 0.006	0.936 ± 0.025	+0.0004	1.0000

For the high-entropy alloy, valence-electron weighting produced the lowest mean MAE and significantly improved over atomic-number weighting. Covalent-radius weighting gave the lowest nanoparticle MAE, but the paired difference was not significant. Atomic-number weighting remained the lowest-MAE encoding for the Materials Project subset, and none of the alternative encodings differed significantly after correction.

Validation partitions and chemical generalization

The curated Materials Project subset contains 250 structures. The random 80/20 protocol therefore uses 200 training and 50 test structures. The composition-only baseline contains five elemental-fraction channels, and the leave-one-chemical-system-out analysis contains ten chemical-system groups, as shown in Table S5. These grouped tests are used to probe chemical generalization rather than to claim exhaustive coverage of Materials Project chemical space.

Table S5: Generalization performance under random, reduced-formula, and chemical-system holdout protocols. Values are mean ± standard deviation. "Train/test" gives the number of structures; ranges are shown for the formula-group splits. Random and formula protocols used 20 partitions, whereas chemical-system generalization used ten leave-one-system-out partitions. The lowest mean MAE within each protocol is bold.

Split	Partitions	Train/test	Method	Features	MAE	RMSE	R^2
Random split	20	200/50	Composition only	5	0.054 ± 0.007	0.075 ± 0.009	0.893 ± 0.038
	20	200/50	RACE	576	0.113 ± 0.016	0.174 ± 0.028	0.453 ± 0.111
	20	200/50	RACE + composition	581	0.044 ± 0.006	0.062 ± 0.011	0.927 ± 0.034
	20	200/50	SOAP-μ_2	504	**0.042 ± 0.006**	**0.060 ± 0.011**	**0.933 ± 0.021**
	20	200/50	ACE	96	0.096 ± 0.012	0.168 ± 0.026	0.481 ± 0.144
Split by formula	20	175–209/41–75	Composition only	5	0.093 ± 0.028	0.123 ± 0.041	0.651 ± 0.335
	20	175–209/41–75	RACE	576	0.137 ± 0.035	0.210 ± 0.066	0.213 ± 0.250
	20	175–209/41–75	RACE + composition	581	0.064 ± 0.021	0.095 ± 0.045	0.841 ± 0.092
	20	175–209/41–75	SOAP-μ_2	504	**0.056 ± 0.016**	**0.082 ± 0.031**	**0.878 ± 0.053**
	20	175–209/41–75	ACE	96	0.117 ± 0.031	0.192 ± 0.053	0.324 ± 0.278
Split by chemical system	10	225/25	Composition only	5	0.111 ± 0.098	0.136 ± 0.117	0.246 ± 1.206
	10	225/25	RACE	576	0.124 ± 0.113	0.155 ± 0.142	0.246 ± 0.631
	10	225/25	RACE + composition	581	0.097 ± 0.118	0.117 ± 0.142	0.612 ± 0.502
	10	225/25	SOAP-μ_2	504	**0.075 ± 0.079**	**0.098 ± 0.102**	**0.726 ± 0.272**
	10	225/25	ACE	96	0.122 ± 0.105	0.173 ± 0.121	-0.068 ± 0.802

SOAP-μ_2 gave the lowest mean MAE in all three protocols. Relative to RACE, every alternative method in the random and formula-group protocols showed a significant MAE difference after Holm correction ($p_{\text{Holm}} \leq 0.0020$). Under the more stringent leave-one-chemical-system-out protocol, none of the alternatives differed significantly from RACE, reflecting the larger variability among the ten held-out chemical systems.

Computational scalability

Table S6: Compact scalability summary for the 250-structure multicomponent dataset. Generation time and peak resident memory correspond to the representative batch benchmark (three runs); training time is the complete feature-selection plus XGBoost fit time (ten runs). The final two columns are time ratios for five versus one species and for the largest versus smallest atom-count bins.

Descriptor	Features	CSV (MiB)	Generation (s/structure)	Peak RSS (MiB)	Training (s)	Species ratio	Atom ratio
RACE	576	2.98	0.750	118.5	4.07	1.07	1.41
SOAP	11480	27.24	0.220	286.3	24.42	1.05	1.08
SOAP-μ_2	504	2.62	0.196	209.8	3.30	1.03	1.11
ACSF	100	0.25	0.194	201.9	0.52	1.02	1.14
ACE	96	0.43	0.198	159.9	1.05	1.02	1.17

Conventional SOAP had by far the largest native dimension, storage requirement, memory use, and training time. RACE used the lowest peak memory among the five evaluated descriptors and required substantially less storage and training time than conventional SOAP, although its descriptor-generation time was the longest in this implementation. Generation time changed little from one to five species for every method; the stronger atom-count dependence observed for RACE produced a largest-to-smallest-bin ratio of 1.41.

Model and descriptor settings

The principal out-of-fold benchmark used ten repeats of five-fold cross-validation. The supplied source audit identifies a common XGBoost configuration with learning rate 0.05, maximum depth 5, minimum child weight 1, unit subsampling and column sampling, zero L1 regularization, and unit L2 regularization; the random state followed the repeat seed. Feature selection retained 50 variables whenever the native dimension exceeded 50. The chemical-encoding and generalization analyses used 20 repeated random or grouped partitions, and the scalability training benchmark used ten repeats. The RACE scalability implementation used Gaussian widths of 0.08 Å for radial channels and 7° for angular channels. SOAP used a Gaussian width of 0.3, while ACSF used three G2 and two G4 parameter sets. Exact command-line overrides, estimator counts, ACE basis definitions, and all source-level settings are preserved in the machine-readable hyperparameter audit and the original analysis scripts.
